# The Prevalence and Categories of Impacted Maxillary Canines: A Radiographic Study

**DOI:** 10.7759/cureus.40070

**Published:** 2023-06-07

**Authors:** Ebrahim Alshawy

**Affiliations:** 1 Department of Orthodontics and Pediatric Dentistry, College of Dentistry, Qassim University, Qassim, SAU

**Keywords:** panorama, maxillary, canines, impaction, orthodontics

## Abstract

Objectives: The current study aims to identify the prevalence of impacted maxillary canines among the Saudi population in the Qassim region.

Methods: A total of 6,946 panoramic radiographs were collected retrospectively and screened by an experienced orthodontist to determine the frequency of impacted maxillary canines. The Statistical Package for the Social Sciences (SPSS) (IBM SPSS Statistics, Armonk, NY, USA) was used to evaluate the significant differences between categorical variables such as gender and the position of impacted teeth.

Results: Overall, 4,977 patients were included in the final analysis. There were 2,509 (50.4%) males and 2,468 (49.6%) females. The prevalence of impacted maxillary canines in our sample was 2.7%, with a higher prevalence of impacted maxillary canines in males (n=74, 2.94%) compared to females (n=60, 2.43%). The majority of the impacted canines were unilateral (n=105, 78.4%) compared to bilateral (n=29, 21.6%).

Conclusion: Impacted maxillary canines were found in 134 out of 4,977 (2.7%) patients. Males (2.94%) demonstrated a higher impaction rate than females (2.43%). However, the difference was not statistically significant.

## Introduction

One of the most common dental anomalies is the disturbance in the eruption of permanent teeth [1]. The failure of eruption is common in the population, particularly the permanent maxillary canines, and is mainly caused by local factors such as lack of space, retention or ankylosis of primary teeth, and the presence of supernumerary teeth [2,3]. In some cases, impacted teeth can lead to severe complications such as root resorption of adjacent teeth, transposition of teeth, and development of cystic lesions [2,4].

Permanent maxillary canines can be considered as impacted when they do not erupt after root formation is completed or when the canine tooth on the other side has erupted for ≥6 months [5]. Maxillary canines are critical for the mastication process and smile aesthetic since they sit at a very critical angle of the mouth. When upper canines are impacted, the facial aesthetic and teeth function might be disturbed [6].

Maxillary canines are the second most impacted teeth in the oral cavity following third molars. The literature shows that the incidence of impacted maxillary canines in Saudi Arabia ranges from 1.70% to 6.35% [7,8]. Two studies carried out in Riyadh, Saudi Arabia, reported that the prevalence of impacted maxillary canines was 3.37% and 3.41% [9,10]. Another study carried out in Almadinah Almunawwarah, Saudi Arabia, evaluated 14,000 panoramic radiographs and reported a prevalence of 2.51% [11]. Alyami et al. [6] carried out a study to assess the prevalence of impacted maxillary canines in Najran, Saudi Arabia. The authors reported a prevalence of 3.35%. Most of the researchers reported that the prevalence of impacted maxillary canines is more common in females than in males [10,12,13]. Also, the impaction of the maxillary canines occurs 20 times more than the mandibular canines [14,15]. Additionally, the literature suggests that bilateral impacted canines occur less frequently than unilateral canines [2].

Identifying the position of impacted canines is usually carried out by clinical and/or radiographic assessments [16]. Radiographic examination gives a better insight into the impacted tooth and the surrounding tissues. Currently, panoramic radiographs are the most used type in prevalence studies in dentistry [17]. From radiographic views, impacted maxillary canines can be classified according to their position in relation to the midline of teeth, occlusal plane, and lateral incisors [11,18]. Accordingly, impacted maxillary canines can be vertically or horizontally oriented. Also, they can be classified as medially or distally angulated. The more severe the angulation, the poorer the prognosis of the impacted tooth [19,20]. Impacted maxillary canines can be as well classified as buccally or palatally displaced [21].

Providing data on the position and prevalence of impacted maxillary canines will enable health planning officials to deploy appropriate resources to treat them. Accordingly, this study aims to determine the prevalence and position of impacted maxillary canines in the Qassim region.

## Materials and methods

Ethical approval

This study was conducted in accordance with the principles of the Declaration of Helsinki. Ethical approval was obtained from the Committee of Research Ethics, Qassim University (date: November 24, 2021; number: 21-04-19).

Study design

The clinical and panoramic records of 6,946 patients who attended the Dental Clinics at the College of Dentistry, Qassim University, between March 2018 and June 2022 were retrieved for the purpose of this study. We only included Saudi patients with a minimum age of 12 years. By the age of 12 years, the position of permanent maxillary canines can be assessed with panoramic radiographs. Also, early treatment of impacted maxillary canines can start at the age of 12 years [22]. Patients who had orthodontic treatment or were wearing orthodontic appliances at the time of taking the panoramic radiographs were excluded. Also, we excluded patients who had extraction in the upper premolar areas or had missing maxillary canines. We excluded these patients to eliminate any previous attempts of correcting impacted upper canines. Additionally, we excluded patients with systemic or hereditary diseases that might affect the development and eruption of permanent teeth, such as Down syndrome and cleidocranial dysplasia [23].

The permanent maxillary canine was considered impacted if it deviated from the normal path of eruption or was obstructed by adjacent or supernumerary teeth.

We collected the following information from each patient: X-ray date, file number, residential information, age, gender, impacted maxillary canine position, current or previous orthodontic treatment, and hereditary diseases.

Sample size calculation

We used the following formula to estimate the required sample size: $n=(z^2 p(1-p))/d^2$, with n as the required sample size, Z^2^ as the level of confidence (99%) (2.58), P as the estimated prevalence (3.21%), and d as precision (4%).

The previous formula has been suggested to be used in prevalence studies [24]. The assumptions were set at a 99% level of confidence and 0.04 precision. The mean prevalence of the condition was estimated to be 3.21% based on the results of other studies carried out recently in different parts of Saudi Arabia [6,9-11,15,25]. Accordingly, we need to identify at least 130 cases with impacted maxillary canines.

Outcome assessment and statistical analysis

An orthodontist from the Department of Orthodontics, College of Dentistry, Qassim University, examined all the obtained radiographs and entered the data into an Excel spreadsheet (2000) (Microsoft Corp., Redmond, WA, USA). Descriptive statistics, including frequencies, means, and percentages, were calculated using Statistical Package for the Social Sciences (SPSS) version 22.0 (IBM SPSS Statistics, Armonk, NY, USA). The chi-square test was used to assess the statistically significant difference in maxillary canine impaction between males and females. Also, we used it to see whether there were significant differences between unilateral and bilateral impactions and between the right and left canine impactions. A P value of <0.05 was considered statistically significant.

## Results

The clinical data and panoramic radiographs of 6,946 patients were screened initially by an orthodontist. However, only 4,977 patients fit the inclusion criteria, of which there were 2,509 (50.4%) males and 2,468 (49.6%) females. The mean (±SD) age of the study population was 29.8 (±14.9). The demographic data of the included patients are shown in Table 1. The reasons for exclusion are presented in Table 2.

**Table 1:** Demographic data of the included participants

Gender (number (%))	
Males	2,509 (50.4)
Females	2,468 (49.6)
Age (number (%))	
12-22	967 (19.4)
23-32	1,497 (30.1)
33-42	1,094 (22)
43-52	625 (12.6)
53-62	698 (14)
62-73	96 (1.9)
City/governorate (number (%))	
Buraydah	2,278 (45.8)
Unaizah	1,230 (24.7)
Arras	476 (9.6)
Al Badayea	306 (6.2)
Al Mithnab	291 (5.8)
Al Bukayriyah	236 (4.7)
Uyun Al Jawa	75 (1.5)
Al-Mulida	51 (1)
Others	34 (0.7)

**Table 2:** Reasons for exclusion

Reasons (number (%))	
Patients had previous extraction in the premolar area	622 (31.6)
Patients have/had orthodontic treatment	502 (25.4)
Non-Saudi patients	454 (23.1)
Young patients (<12 years old)	374 (19)
Patients with missing canines	17 (0.9)
Patients with systemic diseases	0 (0)

After screening the panoramic radiographs, we identified 151 patients with impacted maxillary canines. We excluded 17 patients for nationality reasons, leaving 134 patients to be included in the final assessment. The prevalence of impacted maxillary canines in the studied population was 2.7%. Concerning the gender of patients, there was a higher incidence of impacted maxillary canines in males (n=74, 2.94%) than in females (n=60, 2.43%) with a male/female ratio of 1.23:1. Although more males were included in this study, this observation did not detect any statistically significant difference (df=1, p=0.238).

**Figure 1:**
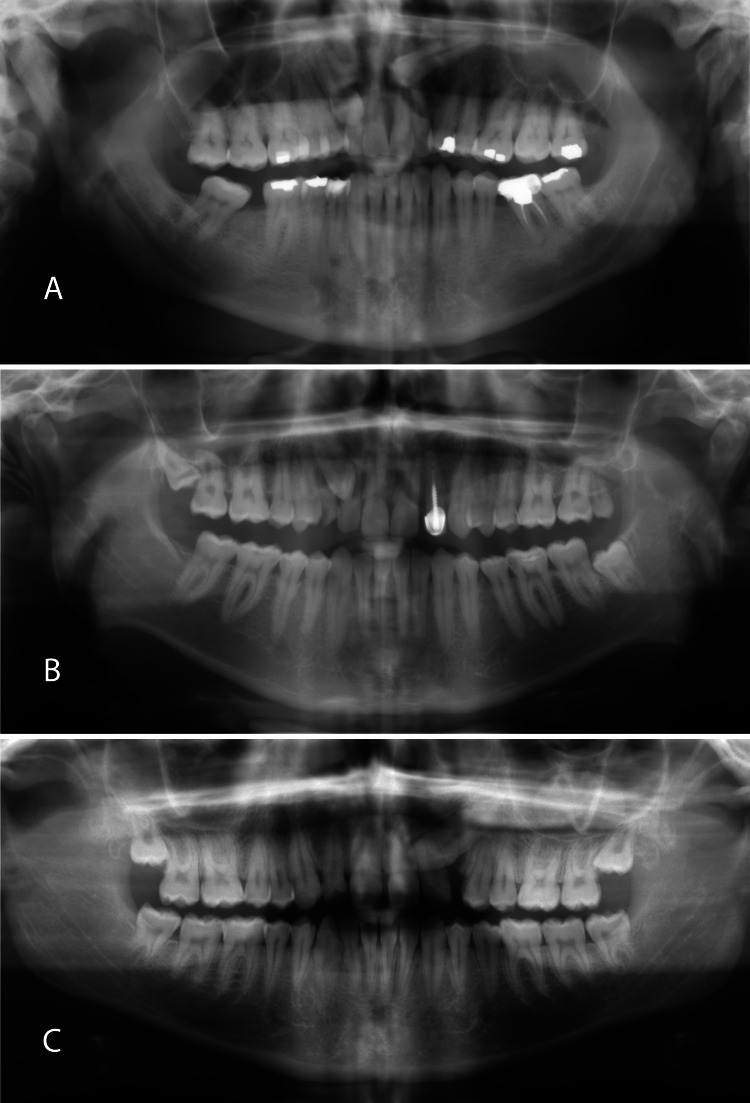
Examples of impacted maxillary canines (A) Bilateral impaction. (B) Unilateral (right side) impaction. (C) Unilateral (left side) impaction.

There was a significant difference (df=1, p=0.001) between unilateral impaction (n=105, 78.4%) and bilateral impaction (n=29, 21.6%). The chi-square test showed no significant differences between the right and left impaction (df=1, p=0.900). However, there were significant differences detected between the right side and bilateral impaction (df=1, p=0.001) and the left side and bilateral impaction (df=1, p=0.002). Figure 1 shows examples of impacted maxillary canines according to their occurrence and position. The distribution of impacted maxillary canines according to gender and position is presented in Table 3.

**Table 3:** Distribution of impacted maxillary canines according to gender and position

Position	Gender		Total (number (%))
	Male (number (%))	Female (number (%))	
Right	29 (21.6)	24 (18)	53 (39.6)
Left	27 (20.2)	25 (18.6)	52 (38.8)
Bilateral	18 (13.4)	11 (8.2)	29 (21.6)
Total	74 (55.2)	60 (44.8)	134 (100)

## Discussion

Unerupted maxillary canines can significantly deteriorate the function and aesthetics of the affected patients. The treatment of impacted canines can be difficult in some situations, and it might involve surgical procedures. The current study showed that the prevalence of maxillary canine impaction among the Saudi population in the Qassim region was 2.7%.

Panoramic radiographs were collected from the dental clinic at Qassim University as it accepts and treats patients from different parts of the Qassim province. It also provides a wide variety of people and covers the whole region. We carried out a sample size calculation using a valid formula utilized in prevalence studies to ensure highly accurate findings [24]. We were able to achieve the minimum required sample size at a 99% level of confidence and 0.04 precision. The sample size calculation is necessary to establish accurate and precise findings of disease prevalence among the targeted group [24]. Non-Saudi patients, patients with hereditary diseases, and patients wearing or who had previous orthodontic appliances were excluded to reduce the introduction of bias to our findings. Including patients from outside Saudi Arabia would affect our conclusions as we found 17 non-Saudi patients with impacted maxillary canines.

Several studies have been published assessing the prevalence of impacted maxillary canines among the Saudi population in different parts of Saudi Arabia. Melha et al. [10] carried out a study in Riyadh and reported that impacted maxillary canines were found in 79 patients with a ratio of 3.37%. This is supported by another study that reported that the incidence of impacted maxillary canines in Riyadh was 3.41% [9]. Although the main aims of the previous two studies were to evaluate the prevalence of impacted upper canines among the Saudi population in Riyadh, they both failed to mention the exclusion of non-Saudi patients. Also, Haralur et al. [9] did not exclude patients who had previous orthodontic treatment, which might have significantly affected the accuracy of their findings.

Another study carried out in Almadinah Almunawwarah reported a prevalence of 2.51% of impacted maxillary canines [11]. Although they included a considerable number of patients (14,000), they did not carry out a sample size calculation nor excluded non-Saudi patients. Accordingly, the precision and accuracy of their result might significantly be affected.

Other studies were carried out in different parts of Saudi Arabia and reported that the occurrence of impacted maxillary canines was between 1.70% and 5.35% [6,8,15].

Unfortunately, most of the studies that were performed in Saudi Arabia did not mention the exclusion of non-Saudi patients. Only one study reported that they included Saudi people in their final assessment [13]. In our study, it was necessary to exclude non-Saudi patients to ensure that the obtained findings represent the Saudi population in the Qassim region. Our finding would have changed from 2.7% to 3.03%, as we identified and excluded 17 non-Saudi participants with impacted maxillary canines. Furthermore, only two studies of the previously mentioned studies carried out a sample size calculation [6,13].

The findings of the current study showed that the prevalence of impacted maxillary canines was higher in males than in females. In contrast, in the study by Haralur et al. [9], the prevalence of impacted maxillary canines was reported to be higher in females than in males. Haralur et al. [9] included 8,517 patients in their study, but they did not describe the distribution of male and female participants. Another study also stated that the occurrence of impacted maxillary canines was significantly higher in females (73.7%) than in males (26.3%) [15]. However, the authors of the previous study included a significantly higher number of female patients in their study (62.66%). Accordingly, the occurrence of impacted maxillary canines will be expected to be higher in females as they included more female patients compared to male patients in their final assessment.

The present study showed no significant differences between the maxillary right (n=53) and left (n=52) impacted canines. Al-Ramil et al. [8] found that canine impaction occurred at a similar rate between the right and left sides. Alyami et al. [6] reported a slightly higher prevalence of maxillary canine impaction on the left side (n=36) compared to the right side (n=31). Other studies also stated that upper canine impaction occurred at a higher rate on the left side compared to the right side [11,13,15].

The current study provides valuable information about the prevalence of impacted maxillary canines among the Saudi Arabian population in the Qassim region. We carried out this study to fill the gap and provide more accurate information about the incidence of maxillary canine impaction in the Qassim region.

Study limitations

We retrieved the clinical and radiographic records from a single dental clinic. It would be better to recruit patients from multiple dental clinics for generalizability and accuracy purposes. However, the included patients in this study came from 10 different places in the Qassim province, which might improve the accuracy and generalizability of our findings. Although the study was carried out retrospectively, we do not think that our current findings would change significantly if it was carried out prospectively. However, caution must be undertaken when generalizing the outcomes of the study. In the current study, more males (50.4%) were included and assessed compared to females (49.6%). The prevalence of impacted maxillary canines for both genders would be more accurate if the number of the included males and females were exactly similar.

## Conclusions

The current study identified 134 out of 4,977 (2.7%) patients with impacted maxillary canines. Impaction occurred more in males than in females, although there was no significant difference. A higher incidence of impaction was found unilaterally (78.4%). Canine impaction occurred at a similar rate between the left and right sides.
